# Longitudinal Dynamics of HPV16 Antibodies in Saliva and Serum among Pregnant Women

**DOI:** 10.3390/v14112567

**Published:** 2022-11-20

**Authors:** Tiina Pirttilä, Stina Syrjänen, Karolina Louvanto, Vuokko Loimaranta

**Affiliations:** 1 Institute of Dentistry, University of Turku, 20520 Turku, Finland; 2 Department of Pathology, Turku University Hospital, 20520 Turku, Finland; 3 Department of Obstetrics and Gynecology, Faculty of Medicine and Health Technology, Tampere University, 33100 Tampere, Finland; 4 Department of Obstetrics and Gynecology, Tampere University Hospital, 33500 Tampere, Finland

**Keywords:** human papillomavirus, oral, HPV16, saliva, serum, IgA, IgG

## Abstract

Oral infections with high-risk (hr)HPV genotypes are associated with a subset of head and neck squamous cell carcinomas. Oral hrHPV infections may result from having oral sex, but also from horizontal infection from mouth to mouth. In such cases, saliva can serve as a vehicle for HPV transmission. Still, the prevalence and dynamics of salivary HPV antibodies in healthy non-vaccinated individuals are poorly known and the role of the salivary antibodies in protection from oral HPV infection is unclear. We used an ELISA assay to evaluate the dynamics and correlation of oral HPV16 infection and HPV16L1 and E7 specific antibody levels in saliva and serum samples among 39 women, 13 of which had persistent oral HPV16 infection. The women were mothers-to-be, sampled before delivery and followed up for 36 months postpartum. HPV16L1 IgG and sIgA antibodies were regularly detected in saliva. Antibody levels in serum remained stable during the 36-month follow-up, while antibody levels in saliva fluctuated. There was considerable individual variation in salivary HPV16L1 antibody levels, and some women had persistent oral HPV16 infection but no salivary antibodies. No differences in salivary HPV16L1 levels were found between the women with persistent or transient oral HPV16 infection.

## 1. Introduction

Persistent infection of genital mucosa with high-risk human papillomaviruses (hrHPVs) of alpha genera is the main etiological agent of cervical intraepithelial neoplasia and carcinoma. Persistent hrHPV infections are also associated with other neoplasms, including a subset of head and neck squamous cell carcinomas (HNSCC), especially oropharyngeal squamous cell carcinomas (OPSCC) [1,2]. Currently, the incidence of OPSCC (especially among young adults) has increased tremendously. The importance of hrHPV in these OPC cases has significant geographic variation, between 19 and 70%, being highest in many developed countries [3]. Mucosal HPV infections are highly common, and nearly all will acquire the infection during their lifetime. Mucosal hrHPV infections are often regarded as sexually transmitted, but also vertical or horizontal (mouth-to-mouth) transmission of oral HPV infections is suggested [2,4]. Additionally, children can be infected at an early age and transmission from mother to her child may continue during early childhood, [5]. The maternal oral cavity is a likely source of HPV virions and maternal saliva one of the vehicles of transmission.

The immune system manages to clear most of these infections, but some escape the immune clearance and remain persistent, and the virus can even integrate into human genome. Persistent hrHPV infection has shown to be the mandatory step for malignant transformation of infected epithelial cells [2,6]. HPV infections induce both humoral and cell-mediated immune responses. Naturally induced IgG and IgA antibodies against HPV16 capsid proteins are commonly found in human serum [7]. Systemic serum responses are well-studied, and the IgG response is generally long-lasting and associated with cumulative exposure to the virus, while IgA responses decay faster [7,8,9]. However, the local antibody responses are less studied. Some studies have shown local sIgA production to follow genital HPV infection in cervicovaginal secretions, but results of their association to ongoing infection are contradictory [10]. Even less is known of salivary sIgA responses to oral HPV16 infection. In cross-sectional studies antibodies, both IgG and sIgA/IgA, against HPV16 are reported in the oral cavity [11,12,13,14,15]. However, most of these studies have explored oral fluid samples containing mainly oral mucosal transudate and gingival cervicular fluid meaning that these HPV antibodies originate mostly from serum [16]. In saliva, sIgA is by far the most abundant antibody type and over 90% of IgA normally found in saliva is locally produced and secreted into saliva as sIgA [16]. HPV16-specific antibodies in saliva are measured only in two studies [11,12], and they both show weak/no correlation between saliva and serum antibody levels. Because no longitudinal studies exist, the role of salivary antibodies in the protection from oral infections remains unknown.

In this study, we aimed to explore the prevalence and dynamics of salivary HPV16 antibodies in accordance with oral HPV16 DNA positivity and antibody responses in sera among mothers-to-be in a longitudinal setting of 36 months. Knowledge of the natural history of asymptomatic oral HPV infections and salivary antibody responses in mothers increases our understanding not only of oral HPV16 infections in women but also of the natural dynamics of HPV infections in families.

## 2. Materials and Methods

### 2.1. Study Population

The participants of this study were 39 pregnant mothers, selected from the cohort of 329 women of the Finnish Family HPV Study, based on their previously described oral HPV status during the first three years follow-up [17,18]. Demographic data from the structured questionnaires at baseline and at 3-year visits were used.

### 2.2. Sample Collections

Oral and genital brush samples were collected from pregnant mothers at the 3rd trimester of pregnancy, and 2, 6, 12, 24 and 36 months postpartum [17,18,19]. For HPV testing, DNA was first extracted from scrapings using the high-salt method follows by nested PCR to amplify HPV DNA (MY09/MY11 and GP05+/bioGP06+ as external and internal primers). HPV genotyping was performed by using the Multimetrix Kit (Progen Biotechnik GmbH, https://www.progen.com), which detected 24 HPV genotypes as described earlier [18]. The study was accepted by the Ethics Committee of the Intermunicipal Hospital Southwest Finland (#3/1998, #2/2006).

Serum and whole saliva samples were collected at the same visit, aliquoted and stored at −70 °C until used. Before storage, the blood samples were centrifuged at 2400× *g* rpm for 10 min (Sorvall GLC-2, DuPont Instrument, Newtown, CT, USA), and divided into three aliquots. A protease inhibitor aprotinin (Sigma-Aldrich, Merck, Darmstadt, Germany) or cOmplete EDTA-free (Roche, basel, Switzerland) was added to the saliva samples or dilution buffers when saliva samples were diluted. Before analysis, the saliva samples were thawed and centrifuged 5 min at 13000× *g*, and the supernatant was used for analysis.

### 2.3. Anti-HPV16 Antibody Measurements

All anti-HPV16L1 antibodies were measured using a commercial HPV16L1 kit (Cusabio Biotech Co., Ltd., Houston, TX, USA). The serum IgG was measured according to the manufacturer’s instructions, and the protocol for serum IgA and saliva IgA and IgG measurements was modified as described earlier [12]. Briefly, before measurement, the saliva samples were diluted at 1:3 (IgG) or 1:10 (IgA) in the dilution buffer provided in the kit. Serum samples were diluted at 1:1000 for IgG and 1:100 for IgA measurements. The HPV16L1 kit contained the secondary IgG antibody which was used in saliva and serum IgG assays, while for IgA detection the secondary antibody was replaced by anti-human IgA—HRP conjugate (Dako, Copenhagen, Denmark, 1:5000 dilution in buffer provided in the kit). The color formed from the TMB substrate was measured at A450 nm. The results were expressed as measured absorbance (Au). All samples were analyzed at least in duplicates.

For anti-HPV16E7 antibody measurements, an ELISA assay was set up. High-binding microtiter plates were coated with 1 µg/mL of recombinant E7 protein (ProteinX Lab, San Diego, CA, USA) overnight at +8 °C. The wells were washed three times with phosphate-buffered saline (PBS, pH 7.2) and blocked with 5% bovine serum albumin (BSA). After incubation for one hour at room temperature (RT, 21–23 °C), 100 µL of serum samples were added to each well (dilution of 1:100 for IgG and 1:10 for IgA) and incubated for one hour at RT. The wells were then washed three times with PBS and HRP conjugated anti-human IgG (Dako, dilution 1:5000), or IgA (Dako, Copenhagen, Denmark dilution 1:2000) was added. After one hour of incubation followed by three washes, the color formed from the TMB substrate was measured as given above. All samples were analyzed at least in duplicates.

### 2.4. Statistical Analysis

In total, 13 mothers with persistent oral HPV16 DNA detection for at least 12 months or longer (range 12–27 months) were selected. In addition, 26 mothers with only transient oral HPV16 DNA positivity were included (0–3 positive oral HPV16 DNA samples during the follow-up), who remained HPV16 negative at least for 24 months. All statistical analyses were run using the IBM SPSS software (version 25.0.0.1, IBM, NY, USA) Differences between the groups were evaluated with one-way ANOVA. Correlation coefficients were calculated with a nonparametric Spearman’s rho assay. Results were considered significant at *p*-values of 0.05 and under

## 3. Results

### 3.1. Study Population

Of selected 39 mothers, 13 had persistent and 26 had transient oral HPV16DNA infection. Not all mothers attended all follow-up visits. The number of samples at each time-point and their HPV16DNA positivity are shown in Table 1. 

The mean age of mothers selected in the oral HPV16 persistent group was 25.3 years (range: 18–29) and in the transient oral HPV16 infection group, 27.0 (range 19–39). There were no significant differences in smoking, alcohol consumption, usage of oral contraceptives, sexual habits or number of lifetime sexual partners (Table S1).

### 3.2. HPV16L1 and HPV16E7 IgG and IgA Antibodies in Saliva and Serum

There was a wide individual variation in anti-HPV16 antibody responses (Table 2). No significant difference was found between those with persistent and those with transient oral HPV16 infection in any tested antibody types.

A moderate correlation was found between serum values of HPV16 E7 and HPV16 L1 (Spearman’s rho 0.348, *p* < 0.01 for IgG and 0.445, *p* < 0.01 for IgA). If values median + 2xSD were considered as elevated responses for HPV16E7, one participant had more than one sample with elevated serum antibody levels of both HPV16E7 IgG and HPV16E7 IgA. In addition, three other women had more than one elevated HPV16E7 IgG only and one HPV16E7 IgA values. Two out of these five women belonged to the persistent group.

HPV16 L1 IgG antibody levels in saliva and serum samples collected at the same visit showed a moderate but significant correlation (*ρ* = 0.276, *p* < 0.01). In the more detailed analysis, we found that saliva and serum IgG values correlated best in the samples collected during pregnancy (*ρ* = 0.395, *p* < 0.05). No correlation was found between saliva and serum HPV16L1 IgA levels (*ρ* = −0.013).

### 3.3. Dynamics of Salivary Antibodies

**Figure 1:**
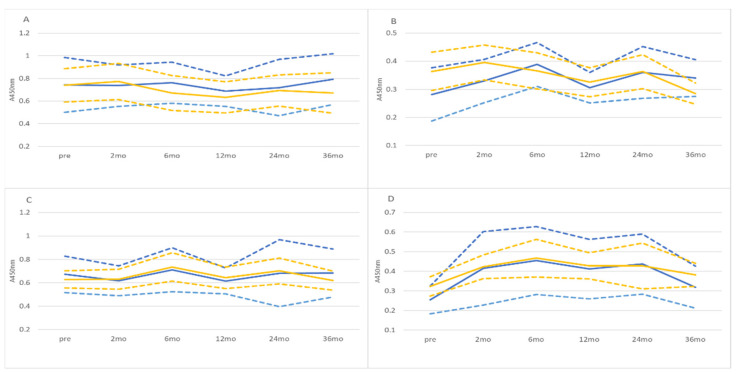
Dynamics of saliva and serum HPV16 L1 antibodies of women who had either persistent (blue lines) or transient (yellow lines) oral HPV16 infection. Mean: solid line, 95% confidence, upper and lower: dashed line. (**A**) saliva sIgA, (**B**) saliva IgG, (**C**) serum IgA, and (**D**) serum IgG.

**Figure 2:**
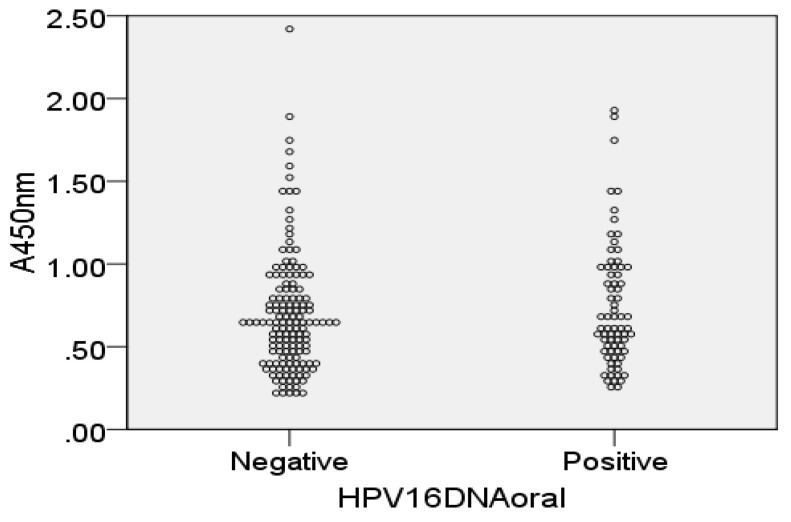
IgA levels in saliva samples that were collected at the time point when HPV16 DNA was detected (positive, *n* = 73) or not detected (negative, *n* = 144) in the oral cavity.

The dynamics of anti-HPV16L1 antibody responses during the follow-up are shown in Figure 1. No significant differences were seen between follow-up visits, except for serum IgG levels which were significantly (*p* < 0.05) lower during pregnancy than postpartum in both groups. No statistically significant differences were noted between individuals with persistent (oral HPV16DNA detected >12 consecutive months) or transient (oral HPV16DNA negative ≥24 consecutive months) oral infection. Furthermore, cross-sectional comparison of all saliva samples with current oral HPV16 DNA positivity to the HPV16DNA negative samples revealed no difference in salivary antibody levels (Figure 2 (sIgA), (IgG) not shown).

**Figure 3:**
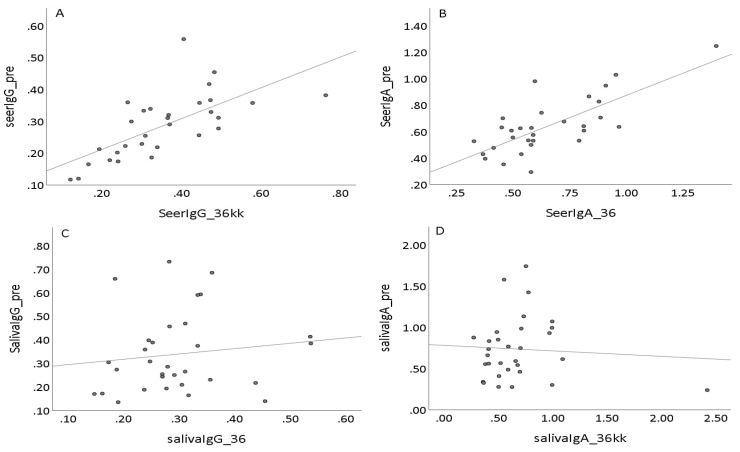
Correlation of serum anti-HPV16L1 IgG (**A**), anti-HPV16L1 IgA (**B**), saliva anti-HPV16L1 IgG (**C**), and anti-HPV16L1 IgA (**D**) levels of the same individual at different time points. The Ig values were measured from saliva and serum samples collected during pregnancy and compared to values measured from samples collected 36 months after delivery.

As expected, the serum HPV16L1 IgG antibody levels appeared to be rather stable, i.e., the serum IgG values measured before delivery correlated well with values measured from the same individual after three years of delivery (Figure 3A, *ρ* = 0.741, *p* < 0.01). Similar correlation was also found in serum HPV16L1 IgA-antibody levels measured during pregnancy and three years later (Figure 3B, *ρ* = 0.699, *p* < 0.01). Salivary HPV16L1 antibodies, whether sIgA or IgG, were less stable. There was no significant correlation between antibody levels in samples taken during pregnancy and three years later after delivery (Figure 3C,D, *ρ* = 0.209 and 0.204, respectively, *p* > 0.2). However, HPV16L1 IgG and sIgA antibody levels in saliva taken at 12-month and 36-month visits correlated significantly with each other (IgG, *ρ* = 0.539, IgA *ρ* = 0.623, *p* < 0.01 for both). Thus, we confirmed that serum HPV16L1 IgG and IgA antibody status is rather stable in sera but found fluctuation in the levels of HPV16L1 specific salivary antibodies.

### 3.4. Individual HPV16 Responses in Saliva and Sera

After we noted a large variation in the salivary antibody responses between individuals even with similar HPV16DNA findings, we decided to explore the individual responses in more detail.

**Figure 4:**
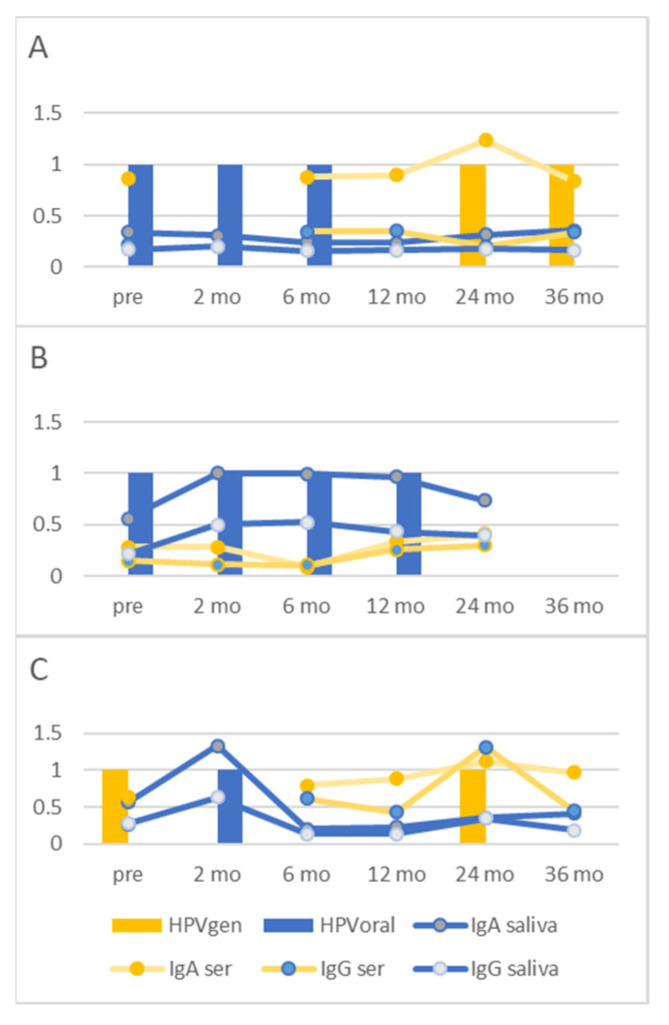
(**A**–**C**) Individual saliva and serum (s)IgA/IgG HPV16L1 responses to oral and genital HPV16 infections. The columns represent genital (HPVgen) and oral HPV16DNA (HPVoral) DNA positivity at different time points, and the lines show the levels of serum and saliva antibodies, as indicated.

Among these, an interesting finding was that in some individuals, even repeated/long-lasting oral HPV16 infections induced only negligible salivary sIgA responses, as shown in Figure 4A. We have earlier characterized the physical state in oral HPV16-persistent women, and based on that data, the present individual had both episomal and integrated HPV16 in her oral sample. We could also identify cases where oral HPV16 infection induced salivary sIgA response but no change in serum IgA/IgG levels (Figure 4B). Additionally, in contrast to common findings, one individual had a fast serum IgG kinetics where IgG levels decayed within 12 months, and saliva antibody responses even faster (Figure 4C).

## 4. Discussion

This is the first study, to our knowledge, where the dynamics of natural oral HPV infection and salivary antibody responses are evaluated in a longitudinal setting. We found that HPV16L1-specific antibodies were equally common in saliva as in the serum taken from unvaccinated individuals, but the antibody levels in saliva fluctuate more than in serum.

We found a moderate but significant correlation between HPV16L1 specific IgG antibodies, but not for IgA/sIgA antibodies, in serum and saliva, which is in line with our earlier study with male participants where no correlation was found between serum and saliva anti-HPV16 IgA levels [12]. Salivary IgGs are mostly serum-derived via mucosal surfaces and gingival fluid while sIgA, the main immunoglobulin type in saliva, is locally produced [16], which is a likely explanation for the difference in correlation. The highest correlation between saliva and serum IgG levels was found during pregnancy, which is in line with the fact that gingival inflammation is highly prevalent during pregnancy, resulting in increased exudation of gingival fluid, and thus serum-derived IgG, into the oral cavity [20,21]. Still, similar correlation between serum and saliva anti-HPG IgG levels are reported also in non-pregnant HIV-positive cohort [11].

In general, the serum anti HPV16L1 IgG levels were significantly lower during pregnancy than after delivery and later during the follow-up. This follows the pattern reported for the total serum IgG levels and suggested to result both from immune suppression and hemodilution [22,23,24]. Interestingly, HPV-specific serum IgA levels did not follow the same pattern. The biological meaning of this phenomenon requires further studies. The literature of the total IgA levels in serum during pregnancy is contradictory, both decrease and no change during the third trimester compared to non-pregnant women are reported [23]. In saliva, the total IgA levels increase during late pregnancy and lactation, but no increase was reported in specific IgA responses in an old study related to oral streptococci [25]. Accordingly, no significant differences between time points was seen in salivary anti-HPV16 sIgA levels in the present study.

Serum HPV16-specific IgG responses are reported to be relatively stable, while IgA is suggested to be more related to recent or ongoing infections and revert within 12 months [7,8,9,26]. In our study group, both HPV16L1 IgG and IgA levels in serum were relatively stable during the 3-year follow-up. Interestingly, we could identify one individual among the 33 women studied, who developed a quick IgG seroresponse to HPV16 infection which waned within 12 months. Thus, low serum IgG levels may not necessarily mean sparse exposure to the virus.

Most individuals had more than one HPV16DNA positive sample and these repeated or re-activated infections may explain that no difference was seen in salivary IgA levels between samples from current and cleared oral HPV16 infections. In addition, our earlier results suggest that no significant difference exists between mean copy number of HPV16 in oral asymptomatic infections that either persist or are cleared [27].

The capacity of naturally induced antibodies to protect against HPV infection is under debate and the results are contradictory. Earlier reports have suggested that low levels of total salivary sIgA may correlate to persistent oral HPV infection [28], but this has not been confirmed by other studies [29]. Furthermore, our previous study from the Finnish Family cohort showed that individuals who cleared their HPV16 infection had the highest serum IgG HPV16L1 antibody levels, and the lowest levels were found in individuals who acquired incident genital HPV16 infection [30], but also contradictory results exist (e.g., [31]). In our small study population, we did not find any significant differences between salivary or serum HPV16L1 specific responses in persistent or transient oral HPV16 infection subgroups. Instead, we saw considerable individual differences in salivary (and serum) HPV16L1 antibody responses to oral HPV16 infections. Furthermore, our current results suggest that oral HPV16 infection induces local salivary sIgA responses not necessarily seen in serum antibody levels and, on the other hand, infections on other mucosal surfaces (at least on genital mucosa) do not necessarily induce responses seen in saliva.

We found high levels of serum HPV16E7 antibodies in 13% of our study population. This is similar to an earlier report where 11% (178/1599) of healthy individuals were E7 seropositive [32]. In a recent meta-analysis, serum antibodies against HPV16 early proteins, especially E6, but also E7, were estimated as biomarkers for HPV-positive oropharyngeal cancer [33]. For example, in a large screen of 1496 cancer cases and 1425 controls, anti-HPV16 E7 antibodies in serum were associated with cancer of the upper aerodigestive tract, especially oropharyngeal cancer [34]. In individuals without HPV-related cancer, serum antibodies to HPV16E7 could not be associated with baseline oral HPV16 DNA prevalence or oral HPV16 persistence [31]. This was true also in our study, where repeatedly high serum HPV16E7 antibody levels were detected in individuals with varying oral HPV16DNA status, and no difference was seen in HPV16 E7 antibody levels between persistent and transient HPV16 infection groups.

Children may acquire their first oral HPV infection already at an early age, and even as a pre- or neonatal infection [35,36,37]. However, we have recently shown results suggesting that transmission from a mother to her child continues during early childhood [5]. The maternal oral cavity is a likely source of the transmitted HPV, and, on the other hand, maternal salivary antibodies might prevent this transmission and protect the child from an early horizontal infection. In our small cohort of mothers-to-be, we found considerable individual variation in salivary HPV16L1 antibody responses, and some women had oral HPV16 infection but no salivary antibodies. If saliva is considered as one of the vehicles of mother-child HPV transmission, these mothers do not provide protective salivary antibodies to their offspring but may still predispose them to virus particles in saliva. Whether or not this creates an increased risk of maternal HPV transmission remains to be studied.

Salivary flow rate is an important modifier of the concentration of all components, including antibodies, in saliva. The samples were collected at clinics where the salivary flow rate was not possible to record. Thus, the comparison of antibody levels without knowledge of the flow rate contains a risk of bias. Salivary flow rate can, however, be considered to be rather stable in healthy young adults, making comparisons between time points and within an individual less affected by variation in the flow. The weakness in our study is that we could not define a cut-off value for the positive and negative antibody levels in saliva or serum IgA due to lack of reliable negative controls. This restricts comparison with previous serological studies where only seroconversions are reported.

## 5. Conclusions

To conclude, our results show that anti-HPV16 antibodies are commonly found in saliva, and they differ from serum responses. In saliva, the antibody levels are dynamic, and no difference was found in salivary antibody levels between women with persistent or transient oral HPV16 infection. Further studies are needed to show the biological importance of salivary antibodies in clearance of oral HPV16, and their possible role in viral mouth-to-mouth transmission.

## Figures and Tables

**Table 1:** Number of samples at different time-points in persistent * and transient ^#^ oral infection groups.

					HPV16 DNA Positive ^&^			
	Serum Samples		Saliva Samples		Genital		Oral	
	persist	trans	persist	trans	persist	trans	persist	trans
pre	11	26	11	26	1	2	4	7
2 mo	10	23	10	25	0	1	7	12
6 mo	13	26	12	26	0	0	7	7
12 mo	12	23	13	23	5	6	9	4
24 mo	9	21	12	26	7	12	9	3
36 mo	10	22	10	23	8	12	6	2

* oral HPV16 DNA positive samples > 12 consecutive months. ^#^ oral HPV16 DNA negative samples ≥ 24 consecutive months. ^&^ Rautava et al. [17]; Louvanto et al. [18].

**Table 2:** The ELISA-values (A450) of saliva and serum samples from individuals with persistent or transient oral HPV16 infection, min-max (median). Note different dilutions in different assays.

	Saliva sIgA *	Saliva IgG ^#^	Serum IgA ^&^	Serum IgG ^
***HPV16L1***				
*persistent*	0.24–1.74 (0.66)	0.13–0.69 (0.31)	0.09–1.55 (0.65)	0.11–1.10 (0.31)
*transient*	0.20–2.42 (0.63)	0.13–0.93 (0.32)	0.30–1.89 (0.59)	0.16–1.42 (0.37)
***HPV16E7***				
*persistent*	NA	NA	0.08–0.26 (0.19)	0.19–0.56 (0.33)
*transient*	NA	NA	0.11–0.51 (0.17)	0.18–0.61 (0.36)

* saliva dilution 1:10. # saliva dilution 1:3. and & serum dilution 1:100 for L1 and 1:10 for E7. ^ serum dilution 1:1000 for L1 and 1:100 for E7. NA: data not available.

## Data Availability

Not applicable.

## Supplementary Materials

The following supporting information can be downloaded at: https://www.mdpi.com/article/10.3390/v14112567/s1, Table S1. Key demographic characteristics of the participants.
