# Supplementary material for: Longitudinal Dynamics of HPV16 Antibodies in Saliva and Serum among Pregnant Women
Source: Viruses. 2022 Nov 20;14(11):2567. doi: 10.3390/v14112567 (PMC9693129; doi:10.3390/v14112567)
Supplement: Supplementary file 1 [file viruses-14-02567-s001.zip › viruses-2029489-supplementary.pdf]

Table S1. Key demographic characteristics of the participants, not all participants responded to all questions

	Persistent oral HPV16 group (n=13)	Transient oral HPV16 group (n=26)
**Age, mean (range)**	25.3 (18-29)	27.0 (19-39)
**Smoking, current**		
**No smoking**	3	11
**1-10 /day**	5	7
**11-20/day**	3	6
**>20/day**	-	1
**Alcohol use**		
**never**	1	4
**2-3 dose (12g)/week**	1	3
**1 dose/week**	2	9
**1 dose or less/month**	6	9
**Mode of delivery (this pregnancy)**		
**alatie**	10	17
**keisarin leikkaus**	3	9
**Age of first pregnancy (mean ±SD)**	22.8 ± 3.8	23.3 ± 3.5
**Number of given births (mean ± SD)**	1.27 ± 0.47	0.32 ± 0.48
**Age at starting oral contraceptives**		
**never used**	1	1
**14-16**	3	13
**17-19**	6	9
**≥20**	1	2
**Age (years) at first sexual intercourse**		
**≤ 16**	6	17
**> 16**	5	8
**Practice of oral sex**		
**yes**	10	20
**no**	1	5
**Practise of anal sex**		
**yes**	3	5
**no**	8	20
**Number of sexual intercourse/mo**		
**2-4**	5	13
**5-10**	4	9
**>10**	2	3
**Number of lifetime sexual partners**		
**0-2**	4	7
**3-5**	3	4
**6-10**	2	7
**>10**	2	7
**History of STDs (genital warts excluded)**		
**yes**	1	8
**no**	12	18
**History of genital warts**		
**yes**	6	11
**no**	5	14
